# Ten simple rules for providing optimal administrative support to research teams

**DOI:** 10.1371/journal.pcbi.1007292

**Published:** 2019-10-03

**Authors:** Romina Garrido, Casandra A. Trowbridge, Nana Tamura

**Affiliations:** 1 Centre for Genomic Regulation (CRG), The Barcelona Institute of Science and Technology, Barcelona, Catalonia, Spain; 2 Universitat Pompeu Fabra (UPF), Barcelona, Catalonia, Spain; 3 Department of Genetics, Stanford School of Medicine, Stanford University, Stanford, California, United States of America; 4 RIKEN Center for Integrative Medical Sciences, Yokohama, Kanagawa, Japan; Whitehead Institute for Biomedical Research, UNITED STATES

## Introduction

Conducting science nowadays is not only properly devoting time to research but also managing administrative tasks. With increasing amounts of time needing to be invested into these administrative tasks, it leads to less time devoted to science and research itself. According to a *Nature* survey, research group leaders point out having more institutional support for administrative tasks as one of the most important needs they have and that institutions need to provide this support to lab members in addition to the leaders [1,2]. Proper administrative support can have a remarkable impact on the scientific productivity of research groups by contributing to a more efficient and effective work environment. It allows the scientists to focus mainly on the science.

With our experience as administrative-based staff in international research groups located in Japan, Europe, and the United States, we will lay out a number of rules that can provide guidance on where to apply efforts when assisting research teams in academic and research environments. These efforts are typically different from what is experienced when supporting teams in business and industry atmospheres. In research, people are used to freedom of thought and to sometimes challenging authority and questioning rules or any preestablished concepts. You need to be much more patient and flexible, and you don’t get desperate if things are different from what you were used to if you are coming from a different environment. These rules are mainly addressed to administrators directly supporting research teams, such as secretaries, assistants, project managers, research managers, operations managers, or any other similar job position. These roles typically have direct and immediate impact on the daily activity of research groups. These rules may also help the principal investigators and other research leaders understand the contributions and positive impact administrators have within a research group.

## Rule 1: Boost your “soft skills”

You will need a number of technical and specialty skills to perform your daily administrative activities, including “soft skills” [3,4]. Soft skills refer to the more intangible and nontechnical abilities. According to the Research Administration as a Profession (RAAAP) Worldwide project survey, examples of important soft skills are your professional capabilities tied to collaboration, taking responsibility, adaptability, problem solving, and communication [5]. Soft skills influence the manner in which you handle interaction, multitasking, and leadership with a team. These skills are more personality driven and can be a sign of emotional intelligence.

Soft skills come naturally for some people, but these skills can also be learned. Developing your soft skills requires a great deal of people interaction and practice. You can boost these skills by looking for mentors, participating in working groups to hear different perspectives, learning various approaches to tasks, and getting enrolled in training courses and workshops in and outside of your home institution. There are associations that offer educational and skill development resources aimed specifically for research administrators. Some examples include the Society of Research Administrators International (SRAI), ARMA International (formerly the Association of Records Managers and Administrators), European Association of Research Managers and Administrators (EARMA), and the National Council of University Research Administrators (NCURA).

People with strong soft skills are more likely to be successful in research and can contribute to a proficient multilateral research environment.

## Rule 2: Be proactive and be decisive

Adopt a proactive mindset. Tackle challenges and solve problems. Measure a desired outcome and take the initiative to see it through. Taking initiative demonstrates that you are a self-starter, independent, and highly motivated. Being proactive with your work is an opportunity to stand out and excel in your job while increasing the likelihood of stability, progress, and success for your research team and the science being conducted.

It is extremely useful to have the skill of being able to anticipate the needs of the research team, especially the principal investigator. Research teams are very typically busy with managing multiple projects and grants, writing new grant proposals, attending meetings, giving frequent presentations, facilitating lectures, and traveling quite often, among other tasks. Strong coordination efforts and keeping updated common calendars where all these tasks can be visible at a glance will help to keep competing commitments, priorities, and deadlines from overlapping. Helping them stay on time and ahead on tasks is greatly needed. Anticipating what they need ahead of time without being told is invaluable.

Be decisive in your actions. You do not want to overthink the most basic of decisions. Research team members and colleagues need to be able to rely on a person who is assertive and can make confident decisions than to put their trust in someone who is slowed and paralyzed by indecision. When a big decision seems like it could be too much to tackle all at once, break it down, take small steps, get more information, reconsider, and then make the next decision [6].

These characteristics described show your leadership and strategic thinking capabilities and that you are willing to go the extra mile in your work. These qualities are extremely useful to supporting your research team and ultimately help excel the progress of science.

## Rule 3: Be efficient, effective, and communicative

You play a vital role in keeping research teams organized in an efficient and effective manner. This includes helping your team remain mindful of timelines and deadlines, supporting the management of competing tasks, developing engagement strategies, scheduling all necessary meetings, keeping costs minimal, and defining success metrics. It also includes minimizing distractions for yourself as well as for members of the research team. The goal is to have optimal effectiveness and enhance functionality for the whole research team.

Your role greatly helps researchers navigate all the different aspects of administration, including managing requirements, obstacles, and burden. Examples include the following:

- Grants have requirements to fulfill. You can help your principal investigator on the oversight of grant rules and procedures, keeping protocols up to date, writing reports, and fulfilling requests from the Institutional Review Board (IRB).
- Research units with higher bureaucratization have lower scientific performance [8]. You can help identify priorities and action plans, eliminate paperwork and extra processes whenever you can, and avoid pushing out decisions or bottlenecking progress.
- Scientists are sometimes reluctant to accept new administrative procedures and/or to spend funds on administrative activities [7]. You can help ease administrative changes and burden by streamlining and automating tasks whenever possible and independently handling what you can on your own. You can help your principal investigator manage administrative budgets and recommend where funding should be allocated.
- Metrics and indicators of team activities are important in research. Although keeping track of some of the items—such as the number of publications and citations using the Altmetric tool—is relatively easy, it may be more difficult for some others [8]. Important team activities to track include the following: current positions held and who are alumni (to evaluate the success on training) and actions taken for public engagement and communication of science (all forms of scientific communications, which include talks to general audiences, radio and TV interviews, articles in newspapers, blog posts, etc.).

Being efficient and effective cannot happen without communication, communication, and communication. Research administrative roles are typically tasked to manage multiple forms of communication. In addition to being an effective verbal communicator, excellent writing skills are necessary. It is essential to write reports, taking minutes of meetings as well as contacting people by email about a range of issues from funding to organizing a program of lectures [9]. There may be promotional leaflets, course materials, exhibition programs, and prospectuses to be written, proofread, and edited [9].

We encourage you to attend lab meetings, working group meetings, and consortium meetings. Although these meetings may be mainly focused on science and analysis, it allows you to hear updates on projects and gives you the opportunity to engage your research team and ask questions. You can be the point person for identifying best practices and communication plans for administrative tasks, increasing efficiency and effectiveness for the entire research team.

## Rule 4: Collaborate and network with other administrators

Although you are directly supporting researchers and your relationship with them is very important, you also need to nurture the relationship with administrative staff located in other departments of your institution. You will very likely find yourself working with the departments leading finance and grants administration, procurement, events and marketing, information technology, human resources, facilities management, etc. You will need to rely on these departments and their services to successfully streamline needs and solve issues for your research team. In many cases, you are the point person, or the “bridge,” through which all communication funnels between researchers and other departments. Interacting with other members of administration at your institution will also help contribute to creating an efficient work environment, as described in Rule 3.

Not only is it important to build relationships within your institution, but it is also important to do so with external collaborators. This is certainly the case whether you work on a research team that spans across multiple institutions or is a part of a large consortium. These types of groups require large-scale coordination efforts, which administrative support professionals are typically tasked to handle. You may find yourself working with administrators from each institution involved in the same study or project as yours. If there are consortium meetings, consider attending them and have administration-based parallel meetings. You can ask the principal investigator to budget for these in grant applications. All these efforts will help you feel like you are part of the scientific enterprise.

Networking can also help your career path in science. This can help us think “outside of the box” and see a bigger picture. Visiting other institutions to learn about how they manage their administrative tasks can be beneficial. It could also lead to new collaborations and other professional opportunities. This “Ten Simple Rules” paper is a great example of the result from professional relationships through networking and collaborations spanning over the last 6 years.

Being able to establish strong collaborations, network, have positive rapport, share resources, and learn from other disciplines are valuable benefits in facilitating your job and coordinating the needs of your research team. This will likely open new doors professionally.

## Rule 5: Be curious about science, even if you do not have a scientific background

When directly supporting scientists working in a particular research field, it can be useful to have some background and exposure in that same scientific discipline to accompany your administrative experience. Although this is ideal for hiring administrators in scientific research fields, it is not easy to find job candidates with such a background, and thus it cannot always be a prerequisite for hiring. When administrators are just starting their career in academia for the first time, they must learn how to adapt to the workplace traditions, customs, and tolerances, especially if they are coming from a career in industry and traditional business in which working standards are very different. Regardless, what is important is for the administrator to be curious about science and make the effort to get a basic familiarity with the scientific discipline and academic working environment they are supporting.

The best way you can learn about the scientific field you are involved with is to have frequent interaction with the scientists you work for. Have scientists explain to you what they do in a nontechnical manner and don’t be afraid to ask questions. Many researchers are used to explaining what they do in layman terms from attending various meetings and giving presentations to audiences with wide-ranging skill sets and educational backgrounds.

We encourage you to get enrolled in basic science or fundamental research courses at a university, whether they are in-person or online courses. Check with your institution on available tuition reimbursement plans or financial assistance programs to enroll in these types of courses. If this type of institutional support does not exist, be proactive about asking your employer to organize these financial assistance programs.

You can also attend scientific meetings, seminars, and conferences aimed at general audiences. Participating in activities where you can interact with people of different professional and educational backgrounds will be very beneficial for you.

You should also take time to learn about your research team’s behaviors and tolerances, as well as your institution’s workplace customs. Academia tends to be more informal than traditional business atmospheres and offers scheduling flexibility, autonomy, freedom to collaborate, and cross-disciplinary thinking. On the flip side, researchers are under immense pressure to be self-starters, continually publish their research, promote and advocate for their work, and find funding sources. Take some time to find how your working style fits into the academic world and how to increase your chances of success.

As mentioned in Rule 1, there are professional associations that offer educational and skill development resources for research administrators—SRAI, ARMA, EARMA, and NCURA. These associations have done extensive work in laying the professional development framework (PDF) and organizing educational opportunities for research administrators. For example, ARMA ran a 12-month project to produce a well-researched and evidence-based PDF for research managers and administrators [10]. From these findings, ARMA developed an outline for professional qualifications and developed courses, certification programs, and online learning opportunities that can contribute to the maturity of the profession, which is very different depending on the regions of the world [11]. Although we focused on the work done specifically by ARMA, joining any of these professional associations will provide guidance on the research administrative career field and give you access to plenty of courses and webinars.

Spending time on getting a better understanding of what’s done in the scientific field you’re involved in will be a very wise and smart investment for you, your research team, and your institution. It also allows you to build a robust and long-term career in the research environment.

## Rule 6: Be responsible with data sharing and handling

Working with principal investigators, research teams, managers, stakeholders, and sponsors means you will be privy to a wealth of information, including highly sensitive and confidential information. This can include exposure to unpublished data and personal health information (PHI). Accessing, using, and/or distributing such sensitive information without permission could give rise to unwanted and serious consequences. Knowing what information to share and with whom is a must, and your discretion needs to be trusted. Make sure to have a clear understanding of when to share the data, with whom, in what format, what security measures are in place, and how the data transfer will be handled. Ultimately, principal investigators, research managers, and institutions are responsible for educating and training employees on the access and handling of sensitive information, as well as providing information on the ethical issues involved. There should be protocols and procedures in place. Be sure to know them and to enroll in any course that can provide you trainings on these matters.

If the study you support works with human subjects and PHI, your research team will be working with the IRB for approval to conduct the study. At times the IRB can feel like an oppressive oversight body bound by regulations and designed to inhibit research, but the IRB is in place to protect human subjects from unethical scientific research while ensuring the highest quality research standards [12]. You can administratively support and soften the relationship between the IRB, the protocol director/principal investigator, and the research team. Administrative roles typically can help with IRB correspondence, protocol modifications and amendment submissions, and preparing reports.

Try to be familiar with the rules and directives governing the sharing of information. Because the principal investigator in your group may not have time to familiarize with the directive, try to investigate how this can affect the science carried out in the group. It will also help your research team maintain compliance.

## Rule 7: Participate in the onboarding process as much as possible

The onboarding process for newcomers is a crucial time for getting them settled into their new position, with their new team, and within the organization as a whole. Although human resources, principal investigators, and lab managers are a large part of the onboarding process for a new employee, administrative staff are typically involved in the process as well. It is important for new employees to feel they are supported by their new organization, especially because starting a new job can cause some challenges. Challenges can be more significant for a newcomer who has taken a job in a foreign country, which is common in research.

Ahead of the new employee’s arrival, connect with the principal investigator and lab manager(s), as well as human resources, to make sure that an onboarding plan is in place with delegation of who will be responsible for each step and task. Providing new employees with a streamlined onboarding process affects all aspects of success for a team and organization as a whole. Examples of action items you can do for new lab members could include the following: being a point person for any questions the new hire may have, assisting with getting the new hire signed up for any relevant onboarding courses or certifications, informing the group about the new hire so they are aware of his/her arrival, and looking for a mentor who can introduce the newcomer to the rest of the lab members and inform him/her about any relevant information. Although all these examples may seem like small tasks and gestures, they can go a long way in making your new hire feels warmly welcomed, valued, and set up for success.

The golden rule to keep in mind is to treat someone the way you would like to be treated, meaning treat a newcomer, at both the professional and personal level, the way you want to be treated if you were in the same situation. You will also have a good working relationship with the new lab member from the very beginning.

## Rule 8: Appreciate and support cultural diversity

There are multiple educational and work opportunities abroad in science and research. It is very common to have research teams with wide-ranging cultural diversity. Expectations for behavior in areas such as leadership, communication, and feedback style can vary across cultures [13]. It is important to be sensitive to cultural differences and to avoid inadvertently stereotyping [13]. Administrative staff, alongside the principal investigator and research management, can actively play a role in learning about the different cultures that exist within the research team and how to avoid misunderstandings and miscommunications. Administrative staff can help define clear expectations for administrative-based actions and tasks while assisting the incoming international team member with overall adaptation with the research team and new work environment.

Culturally related work preferences, such as time management, task orientation, risk orientation, directness, or even sense of humor, have an important impact on the team dynamics. Some internationally based scientists find that the priorities attached to socializing (including the interaction between men and women) differ from what they are used to [13]. They will very likely feel the difference in thoughts and values and often encounter challenges, particularly with the relationship to authority. These views may differ strongly between countries, and you could help the newcomers to adapt to the accepted rules in the institute.

To learn about your team’s various cultural working and social preferences, you can begin by asking them where they are from and institutions they have studied and worked at. Ask about their communication preferences and the management working styles that they are used to when handling tasks with administration, project coordination and management, and overall team interactions and management. You can be the person they can turn to when difficulties arise at work, especially if they feel uncomfortable asking those questions with their lab mates or other colleagues. Encourage them to keep an open mind and ask questions. The goal is to work towards finding a common understanding and establishing clear expectations. In the process, you may end up learning a new method or way of doing something to apply to your own job or with the team as a whole.

You can also recommend ways for them to learn more about the local culture. You can tell them about local traditions and holiday festivities. You can suggest restaurants they should check out and new food they should consider tasting. You can suggest places where they can hear locally appreciated music or museums to visit. Immersion in the local culture is a great way for them to adapt to their new community.

It is important to value cultural diversity and understand differences between people while recognizing that these differences are a valued asset. Multiculturalism improves productivity, creativity, and employee engagement and opens up doors to new opportunities in often unexpected ways [14,15]. Blending and cocreating workflows and methods can help you and the whole team create a rich, balanced, and comfortable environment. Be engaged with the cultural diversity on your research team.

## Rule 9: Treat everyone fairly

On a research team, there are people at very different levels of hierarchy, responsibility, and needs. Administrative staff are typically assigned to give more priority to supporting the requests and needs of the more senior members (faculty, principal investigators, senior postdocs, etc.), but those in earlier career stages (undergraduates, technicians, junior postdocs, etc.) need support as well. Regardless of seniority and status, everyone on the research team needs to feel he/she is receiving adequate administrative support and being treated fairly. They do not all need the same level of support, but the quality of the services you provide to all team members should be similar.

Administrative support resources are not unlimited, so it is good to set a list of services that you can offer to people at each level of responsibility and to review it periodically to make any necessary changes. Do not limit yourself to that list, as you always want to be looking for new ways to expand and grow your skill set. Make sure that it is understood that more senior level requests and urgent matters will always take priority but that you will also address other requests. It is helpful to let people know expectations and timelines related to task completion. Periodically check in with each team member to see if they need assistance with any tasks. Your administrative support benefits the research team as a whole.

People are always the most important asset and should receive excellent service. Everyone should have the feeling that their contributions to the team and to science are important. Once they become alumni, they will be the ambassadors of the institution and of your work.

## Rule 10: Be an active team player and show your unique qualities

Although your contribution to the research team is not scientific, you play a vitally significant role to help the principal investigator streamline the working dynamics of the group. Don’t stay in the shadows or be a mere observer. Work actively to find ways to make administrative procedures easier for your research team. Give your point of view and suggestions for improvements and help develop more effective methods. Engage your research team on their thoughts. Combining both administrative and scientific perspectives when facing problems will inspire you to find creative solutions to support your research team.

Organizations are more than their mission statement, aims, impact, infrastructure, and policies. Organizations are greatly defined by the type of people they hire, which creates the company culture. Show your unique qualities. Let your personality shine through. Be genuine. Everyone in research appreciates character, so be yourself.

Your active team player attitude and unique qualities will have great positive effect on your research team as well as on the institution as a whole. Take pride in that.

## Conclusion

Administrative professionals do more than just assist. They are one of the backbones to successful research and organizations. They have strong skills for multitasking, planning, organization, customer service, and directing large groups of people. They are effective communicators and trusted individuals to handle sensitive information. They are the gatekeepers to senior level individuals and the helpers to the whole team. They are the bridges to other departments and help foster collaborations with other researchers and labs. They take initiative and do what is needed without being told and can anticipate future needs. Their roles in science are constantly evolving, and they continuously take on new tasks. The role of administrative professionals allows scientists to keep their main focus on the science and be less distracted by necessary administrative operations. Research administrators are a valuable asset and deserve professional recognition.

The mantra of many administrative professionals is “No job is too big or too small.” They contribute greatly to the success of research and have a strong impact on science. As Kim C. Carter, SRAI president, says, “In the world of ordinary mortals, research administrators are superheroes” [16].
